# Impact of macro-socioeconomic determinants on sustainable perinatal health care in Portugal: a qualitative study on the opinion of healthcare professionals and experts

**DOI:** 10.1186/s12889-021-10194-0

**Published:** 2021-01-25

**Authors:** Julia Nadine Doetsch, Sandra C. S. Marques, Thomas Krafft, Henrique Barros

**Affiliations:** 1 EPIUnit – Instituto de Saúde Pública da Universidade do Porto (ISPUP), Rua das Taipas 135, 4050-091 Porto, Portugal; 2 Maastricht University, Faculty of Health, Medicine and Life Sciences (FHML), Care and Public Health Research Institute (CAPHRI), Maastricht, The Netherlands; 3 Centro em Rede de Investigação em Antropologia (CRIA) - Instituto Universitário de Lisboa, Lisbon, Portugal; 4 Departamento de Ciências da Saúde Pública e Forenses e Educação Médica, Faculdade de Medicina, Universidade do Porto (FMUP), Porto, Portugal

**Keywords:** Quality of health care, Health care providers, Health personnel, Infant, Premature, Health, Equity, Social determinants of health, Economic recession

## Abstract

**Background:**

The WHO identified the importance of macro-socioeconomic determinants and political context as interlinked key factors affecting healthcare quality and health equity. As a response to the recent economic and financial crisis, Portugal approved in 2011 the Economic Adjustment Programme (EAP) to obtain financial assistance from the Troika in order to reduce public debt. This study aims to analyse the impact of the economic crisis and the EAP on perinatal healthcare quality for very preterm (VPT) and/or very low birth weight (VLBW) infants, as perceived by healthcare professionals and experts, within the health administrative regions of the two major metropolitan areas in Portugal.

**Methods:**

A qualitative approach was applied to receive an in-depth understanding and accomplish perspective variability. A purposive sampling technique was used. Semi-structured interviews were conducted with twenty-one healthcare professionals and experts between October 2018–July 2019. Inductive thematic analysis was performed which encompassed a five-step categorization procedure. Data analysis was undertaken by utilizing Nvivo2011 software. Evolved themes were then associated with WHO’s Quality Standards on Maternal and New-born Care. A framework on the impact of macro-socioeconomic determinants on perinatal health care quality was developed.

**Results:**

Although participants did not perceive the quality of perinatal care had deteriorated, the analysis of their accounts on work experience revealed that it was indeed adversely modified in all WHO Quality Standards. Health care provision was perceived as detrimental in five main areas: 1) Availability of human resources; 2) Functional referral systems; 3) Competent and motivated human resources; 4) Emotional support; and 5) Essential physical resources available. Policy reforms by the EAP resulted in reduced timeliness of care, increased waiting times, cuts in sequence and duration of consultations, and deficiencies in follow-up care for VPT/VLBW infants and their mothers. The EAP directly influenced working environment of healthcare professionals by causing stress, burnout, work absence, and brain drain.

**Conclusion:**

An interrelation between macro-socioeconomic determinants and perinatal health care quality was disclosed. The economic crisis and EAP have adversely modified equitable perinatal health care quality for VPT/VLBW infants and their mothers. Our findings underlined the negative impact of austerity policies on vulnerable populations.

**Supplementary Information:**

The online version contains supplementary material available at 10.1186/s12889-021-10194-0.

## Background

Preterm birth is defined as being born before 37 completed weeks of gestational age (GA). Premature birth has increased worldwide. Each year, one in ten infants is born preterm worldwide which translates into approximately 50,000 preterm births in Europe [1]. Since 2000, preterm birth rates have increased in the European Union (EU) due to demographic changes (e.g., maternal age > 35), changes in lifestyle factors (e.g., higher maternal body mass index), and rise in multiple births related to technological advances (e.g., in vitro fertilization) [2]. In Portugal they have increased even faster than the EU15 average: 7.8% of all births in 2016 were preterm births [3]. Very preterm infants (VPT) are born with less than 32 completed weeks of GA, which commonly signifies to be also born with a low (≤2500 g) or very low birth weight (VLBW) (≤1500 g) [1].

VPT/VLBW infants require comprehensive high quality care as they have a high risk of developing complications such as impaired cognitive ability, long-term morbidity, and mortality [4, 5]. High quality health care is defined as “the extent to which health care services provided to individuals and patient populations improve desired health outcomes by providing safe, effective, timely, efficient, equitable and people-centred health care” and involves equal accessibility to care and equal ability to make adequate use of maternal health services [1]. The World Health Organization (WHO) developed eight Quality Standards (QS) on maternal and new-born quality of care which are categorized into six dimensions: Effectiveness, Appropriateness, Accessibility, Acceptability, Patient-centeredness, and Equity and Safety [6] (Table 1). According to the WHO, skilled and qualified ante-, intra-, and postnatal care can prevent 75% of annual preterm deaths worldwide [6, 7].

**Table 1:** Standards for improving quality of maternal and new-born care in health facilities

Quality Standards [QS]^a^			Quality Statements^b^
Evidence based practices for routine care and management of complications	**QS 1**	Every woman and new-born receive routine, evidence-based care and management of complications during labour, childbirth and the early postnatal period, according to WHO guidelines.	**Quality Statements 1.1a-1.1c** Pre-and Postnatal routine assessments and timely care e.g. Pre-eclampsia, eclampsia, postpartum haemorrhage, reanimation, infections
Actionable information systems	**QS 2**	The health information system enables use of data to ensure early, appropriate action to improve the care of every woman and new-born.	**Quality Statements 2.1-2.2** Pre- and Postnatal standardized medical records, monitoring, analysis feedback provided by health facility
Functional referral system	**QS 3**	Every woman and new-born with condition(s) that cannot be dealt with effectively with the available resources is appropriately referred.	**Quality Statements 3.1-3.3** Appropriate assessed admission, pre-established referred within health facilities, information exchange among HC staff
Effective communication	**QS 4**	Communication with women and their families is effective and responds to their needs and preferences.	**Quality Statements 4.1-4.2** Information on care provision, interaction with staff, coordinated care with information exchange from health and social professionals
Respect and preservation of dignity	**QS 5**	Women and new-born receive care with respect and preservation of their dignity.	**Quality Statements 5.1-5.3** Privacy, confidentiality, informed choices in received services, no denial of services or mistreatment
Emotional support	**QS 6**	Every woman and her family are provided with emotional support that is sensitive to their needs and strengthens the woman’s capability.	**Quality Statements 6.1-6.2:** Option given to experience labour and childbirth with companion of her choice, support to strengthen capabilities during childbirth
Competent, motivated, human resources	**QS 7**	For every woman and new-born, competent, motivated staff are consistently available to provide routine care and manage complications.	**Quality Statements 7.1-7.3:** Access to support staff for routine care with appropriate competences, Health facility has managerial and clinical leadership to undertake quality improvement
Essential physical resources available	**QS 8**	The health facility has an appropriate physical environment, with adequate water, sanitation and energy supplies, medicines, supplies and equipment for routine maternal and new-born care and management of complications.	**Quality Statements 8.1-8.3:** Functional, reliable, safe and sufficient facilities, organized pre-and postnatal areas, adequate medicines, supplies and equipment for routine care and management of complications

**Based on:** World Health Organization. (2016). Standards for improving quality of maternal and new-born care in health facilities

^a^Quality standards [QS]: Concise prioritized statement designed to drive measurable quality improvements in the care around childbirth

^b^Quality measures: Criteria that can be used to assess, measure and monitor quality of care

In Portugal, distinct improvements in neonatal intensive care have been recorded since 1980s [8–12] (Table 2). The Directorate-General for Health, a technical and normative body of the Ministry of Health, is responsible for guidance and development of public health programmes and aims to improve health care through the provision of national guidelines in Portugal. Low-risk perinatal care consultations are scheduled monthly up to 30 weeks of GA, and biweekly between 30 and 36 weeks of GA at public primary health care centres of the National Health Service (NHS). In the NHS, low-risk pregnancies are monitored by a general practitioner (GP) up until 36 weeks of GA, who records medical examinations in a pregnancy booklet [13, 14]. After 36 weeks of GA and until birth, an appointment is scheduled every 1–2 weeks at the maternity care unit of a public hospital. Private perinatal care is provided by a gynaecologist or obstetrician and paid by the user through out-of-pocket payments, subsystems, or voluntary health insurances. Portuguese law specifies that a preterm infant, which is born with less than 34 weeks of GA, must be delivered in a public hospital with differentiated care functionalities. About 85% of all deliveries occur in public hospitals where pregnant women have free universal access to care [15]. Neonatal care is delivered at three levels: Neonatal intensive care units (NICUs), intermediate care, and nursery. In 2011, 22 NICUs in public and two in private care were registered [11]. In 2014, 24 perinatal care hospitals (HAP) and 17 differentiated perinatal care hospitals (HAPD), mainly concentrated in the central area of the two major metropolitan areas of Portuguese mainland, were listed. In 2019, only 12 HAPD were registered [11].

**Table 2:** Neonatal policies and improvements provided by the NHS in Portugal

Year	Establishment
1980	First neonatal intensive care units (NICUs)
1985	Neonatal Branch of the “Portuguese Society of Paediatrics”
1987	National neonatal transport system and the Nomination of an Experts Committee
1989	National Committee for Women and Child Health
1989	Perinatal Health Care Reform - 9-year programme executed in 3-year steps The reform mainly included:
	a. Reclassification of hospitals into three levels: 1) Level I Coordinating Unit where neither deliveries nor outpatient clinic services are provided 2) Level II Hospitals: - Perinatal care hospitals “Hospitais de Apoio Perinatal” (HAP) for low-risk deliveries 3) Level III Hospitals: Differentiated Perinatal Care Hospitals “Hospitais de Apoio Perinatal Diferenciado” (HAPD) for low and high-risk deliveries in neonatal intensive care units (NICUs) staffed with obstetricians, neonatologists and nurses specialised in neonatology
	b. Closure of hospitals with less than 1500 deliveries per year
	c. Supplying neonatal intensive and intermediate care units
	d. Coordinating units between health centres and hospitals 1) Transport between level II and level III hospitals depending on level of extensive care needed
	e. Specialised training in Neonatology
1990	Post-graduation in Neonatology
1996	National VLBW Network
2000	Mother and Child Hospital Referral Network
2010	Renaming “The Portuguese Society of Paediatrics” to “The Portuguese Neonatal Society”

Macro-socioeconomic determinants such as political context, governance, policies, and economic impact are interlinked key factors, which influence health, healthcare, health equity, and the performance of healthcare systems [1]. In 2011, Portugal approved the Economic Adjustment Programme (EAP) (2011–2014) to obtain financial assistance from the Troika, a decision group formed by the International Monetary Fund, the European Commission, and the European Central Bank, to prevent insolvency [16]. The overall aim of the EAP was to achieve “a balance between re-gaining credibility and debt stabilization, and limiting adverse impacts on growth”, focussing on three core essentials: short-term financial aid (2011–2014) to fund existing account deficit; fiscal reforms to decrease governmental debt; and structural reforms to enhance Portugal’s growth. The EAP followed fundamental principles of lean government involvement and economic liberalization policies, such as fiscal austerity and reductions in government expenditure.

The specific objective of the EAP for the NHS was to reduce public debt by diminishing waste and stimulating private sector involvement, to economize non-essential health care costs by increasing efficiency, and to enhance pharmaceutical market regulations and hospital management by decreasing contracted budgets (Table 3). Austerity measures and healthcare reforms encompassed budget cuts for NHS healthcare providers, which resulted in reductions in salaries, overtime hour-payments, and retirement benefits. The reforms further induced reorganisation, reallocation, centralization and privatization of services, which led to fundamental changes in the Portuguese health care system [16].

**Table 3:** Key areas of the Economic Adjustment Programme on the National Health System in Portugal

1 Primary care services	**Reinforcement of provision and efficiency of the Primary care services**
	1.1 Equal allocation of general practitioners throughout the country
	1.2 Restructuring of health units into “Agrupamento de Centros de Saúde” and implementing family health units “Unidades de Saúde Familiares”
	1.3 Wages and services associated payments
	1.4 Introduction of electronic platform of medical records assessed by primary care providers and hospitals
	1.5 Increase of the numbers of USFs to achieve an even geographic distribution of GPs
2 Co-payments	**Increase in NHS co-payments - user fees, “taxas moderadoras”**
	2.1 Revision of the NHS cost-sharing schemes (co-payments) to reinforce primary care usage
	2.2 Automatic indexation to Inflation of co-payment taxes
3 Hospital Care services	**Centralization and Reorganization of public hospitals to attain savings in operational costs**
	3.1 Merging of numerous hospital outpatient services into primary care units
	3.2 Staff reallocation
	3.3 Rationalization of resources and facilities
	3.4 Decrease in staff overtime compensation
4 Pharmaceuticals	**Reduction in public spending**
	4.1 Revision of pricing systems, price reduction in expenditure for Pharmaceuticals
	4.2 Reduction in the regulated price increase rates for pharmacies
	4.3 Reinforcement in compulsory prescription of generic medicine
	4.4 Formation of intensive monitoring mechanisms with evaluation and response to physicians
	4.5 Introduction of clinical guidelines
	4.6 Compulsory electronic-prescription for consistent monitoring evaluation and reporting
5 NHS (General)	**Healthcare cost reduction**
	5.1 Fundamental revision and adjustment of accompanying exemption rules for healthcare payment
	5.2 Reduction in tax allowances for healthcare expenditure by two thirds, including private insurance expenses
	5.3 Revision in provision and purchasing procedures to accomplish savings by centralizing procurement (i.e., reduction in transaction costs)
	5.4 Cuts in non-emergency transportation to healthcare facilities

Based on: European Commission. (2011). The economic adjustment programme for Portugal

We hypothesized that the EAP and the economic crisis affected the occupational environment of healthcare professionals and subsequently their provision of perinatal health care quality. This study aimed to analyse the impact of the economic crisis and EAP on perinatal health care quality for VPT/VLBW infants, as perceived by healthcare professionals and experts, within the health administrative regions of the two major metropolitan areas in Portugal.

## Methods

### Study design and sample

A qualitative approach was applied enabling to receive an in-depth understanding of the depicted problem. A purposive sampling technique was utilized to accomplish variability and balance in perspectives [17]. The study sample (*n* = 21) comprised: i) healthcare professionals (*n* = 14) such as neonatologists, paediatricians, obstetricians and nurses with work experiences in public and private care on prematurity before, during, and after the EAP implementation period; and ii) healthcare experts (*n* = 7) from the fields of politics, economy, sociology and pharmacy, of which some had been involved in health care policy and decision making during the same time period. The majority of healthcare professionals were female (*n* = 11) and the majority of healthcare experts were male (*n* = 6). The age of the participants ranged from 35 to 70 years (Table 4). The two major metropolitan areas of Portugal, Northern region and Lisbon and Tagus Valley, centralize most healthcare units with specialized differentiated perinatal care for VPT/VLBW infants in the country. They were therefore chosen as our study setting.

**Table 4:** Informants information

No	Participant	Profession	Institution of current employment
1	Healthcare professional	Neonatologist	Centro Hospitalar do Porto
2	Healthcare professional	Neonatologist	Maternidade Bissaya Barreto, Coimbra
3	Healthcare professional	Neonatologist	Hospital São João, Porto
4	Healthcare professional	Neonatologist, Peadiatrician	Centro Hospitalar Lisboa Norte, EPE - Hospital Santa Maria
5	Healthcare professional	Neonatologist, Peadiatrician	Pediatrics department at Maternidade Dr. Alfredo da Costa, Lisbon
6	Healthcare professional	Neonatologist	Centro Hospitalar Lisboa Norte, Hospital Santa Maria
7	Healthcare professional	Obstetrician	Hospital São João, Porto
8	Healthcare expert	Obstetrician	Previous: Centro Hospitalar Lisboa Central, Maternidade Dr. Alfredo da Costa
9	Healthcare professional	Obstetrician	Centro Hospitalar do Porto
10	Healthcare expert	Pharmaceutical	Universidade NOVA de Lisboa
11	Healthcare professional	Neonatologist, Peadiatrician	Centro Hospitalar Tamega e Sousa
12	Healthcare professional	Nurse^a^	UCSP Algueirão Sintra
13	Healthcare professional	Nurse^a^	UCSP Algueirão Sintra
14	Healthcare professional	Nurse^a^	UCSP Algueirão Sintra
15	Healthcare professional	Nurse^a^	UCSP Algueirão Sintra
16	Healthcare expert	Economist, Professor	Escola Nacional de Saúde Pública
17	Healthcare expert	Economist, Professor	Nova School of Business and Economics
18	Healthcare expert	Politician, Physician	Parliament
19	Healthcare expert	Politician	National Health Council
20	Healthcare expert	Sociologist	ISCTE-Instituto Universitário de Lisboa
21	Health professional	Obstetrician	Centro Hospitalar Lisboa Central, Maternidade Dr. Alfredo da Costa

^a^specialized in Maternal Health and Obstetrics

### Data collection procedures

Participants were recruited via phone and email. Further contacts for data collection through individual interviewing was ended when inductive thematic saturation was attained [18]. That is, when the team of two researchers involved in collection, processing, and analysis of data agreed that new data tended to be redundant of data already collected and no new topics emerged from latest interviews. The saturation point was achieved after the preliminary analysis of 21 conducted interviews [19]. Data were collected between October 2018–July 2019. A face-to-face, in-depth semi-structured interview technique was chosen to deliver reliable and equivalent qualitative data while benefitting from an in-depth response of the participants [20]. The interviews lasted between 1 to 1 ½ hours.

A paper-based interview guide was developed and pilot tested covering the key questions to be answered within four main areas: a) Current health access and provision; b) Influence of EAP and crisis; c) Policy priorities on prematurity; d) Recommendations (Supplementary material 1). Inquired participants had been previously informed (verbally and in written format) about the study and provided with the interview guide. Participants were not financially compensated for the interviews. They were explicitly notified that the data would be processed as personal opinions of experienced professionals and experts in the subject and time period under study and not as representative of their current employment position and institution. Signed informed consent as well as prior permission for audio recording for analysis purposes was obtained from all participants. Appropriate ethical and consent procedures were taken according to the data protection guidelines of the General Data Protection Regulation (GDPR) [(EU) Regulation 2016/67] and Portuguese law regarding the non-sensitive nature of the data collected from study participants [21, 22].

The interviews were conducted in Portuguese and English language at times and locations chosen by the participants. Interviews were verbatim transcribed, maintaining original connotations, translated into English and verified by the same research team. Though informed on the right of providing insights on their transcripts and translations, none of the participants requested it and thus no transcript was returned to participants. All transcripts were anonymized. Transcripts were stored in a password encrypted file, which is protected in a dedicated storage at the research institution ISPUP and kept for a defined time period of 5 years. Publications and presentations from the study displayed anonymous findings and were subjected to a minimisation of identifiable data.

### Data analysis

A thematic analysis was undertaken utilizing Nvivo2011 software, which provides tools for extracting, arranging, organizing, and comparing significant fragments of the transcriptions in a systematic way. The analysis was performed encompassing a five-step categorization procedure of coding refinement towards the definition of major emerged themes. The coding of the content was checked and matched independently by two researchers. Both researchers agreed upon the application of the same coding scheme (intercoder reliability). The five steps included: Identifying and ranking of key concepts by frequency (Step 1); Sorting ranked key concepts into minor sub-categories called codes (Step 2); Clustering codes into major categories called nodes (Step 3); Clustering nodes to identify major themes (Step 4); and Associating categorized major themes with the eight Quality Standards [QS] on maternal and newborn quality of care by the WHO [6] (Step 5) (Table 5). A graphic flowchart on the interplay between the EAP, the crisis, and the Quality Standards was constructed that emerged during the analysis (Supplementary material 2).

**Table 5:** Visualization of thematic analysis process

Step 1: Key concepts^a^	Step 2: Codes^a^	Step 3: Nodes^a^	Step 4: Themes	Step 5: Quality Standards
experience, nurses, normal pregnancy, medical advice, appointments, preparations, tiredness, immediate referral, neonatologist, waiting, no appointment, EAP, staff, lack, follow, incomplete, risks, aggravated prohibition, direct, negative, hiring, replacing, teams, incomplete, public, retired, public, private	Medical treatment, medical advice, antenatal appointment, postnatal follow-up, prevention, lack of staff, EAP, care provision brain drain, healthcare unit, hospital	Quality, antenatal care postnatal care, Primary care provision, Secondary intra- and postnatal care provision, Waiting times and time management, Psychological and formal support provision	1) Availability of Human resources	QS 1 Evidence based practices for routine care and management of complications
recorded, followed, accompanied, professionals, interest, report, observed, intervention, terms, signed, assist, register, sheet, failure, computer, waiting, EAP, cuts, crisis, GP, schedule, appointments, observe	Observation, Monitoring, data collection, follow-up, systems, EAP cuts, medical records	Monitoring and medical records, Actionable information systems articulation and communication, Physical resources	5) Essential physical resources available	QS 2 Actionable information systems
surgery, manage, request, improvements, sick, essential, concern, terrible, waiting, scientific, coordination, department, diagnosis, send, maternity, unit, closing, staff, lack, EAP, crisis	Staff exchange, hospital merge, closure of maternity units, EAP, crisis, GP, healthcare professionals	Appropriate referral, Shortage in staff and capacity in inter-facilities transport, Non-attendance of antenatal care consultations, Referral system articulation, Gate-keeping-system	2) Functional referral systems	QS 3 Functional referral system
attention, questions, awareness, poor explanation, face, contact, hours, infections, discharge, risks, knew nothing, response, decide, agrees, future, abortion, notion, lie, purpose, lost, horrible, measure, stuck, no information, died	Interaction, Information, knowledge, communication, Information provision, cuts by EAP, salaries, adequate response	Parents and healthcare staff communication, Emotional support provision, Effective communication	4) Emotional support	QS 4 Effective communication
carefulness, important, kind, zero privacy, sense, receive, friendly, involve, approach, expectation, thanks, protect, learned, waited, secure, loving, participation, comfort, staff, lack, EAP, cuts, leave, retire, stress, tired, career, salary, extra, time, working, hours, payment, years, public, private, contracts, nurse, medical doctor, young, cheaper, labour, specialization, job	Accompaniment, comforting care, lack of staff, EAP cuts, emotional support, higher workload, given regulations	Appropriate and respective treatment, Emotional support provision,	3) Competent, motivated, human resources	QS 5 Respect and preservation of dignity
traumatised, guilt, painful, crying, shock, difficult, psychologist, suffer, time lap, lack of feeling, no support, emotions, reasons, alone, reality, obsessive, behaviour, anxiety, panicked, desperate, erase, memory, frightened, staff, cuts, EAP, crisis, emotional, alone, guilt	Emotional management, Stress coping, support, EAP, cuts, psychologist available Observation, premature birth, traumatic event	Emotional and psychological support, Formal and informal support, psychological surveillance	4) Emotional support	QS 6 Emotional support
unfriendly, time, impact, visits, value, knowledge, advised, not motivated, impatient, extreme, avoid, eye-contact, trainee, intensive, staff, complaints, distance, interfere, staff, EAP, cuts, crisis	Healthcare staff, time availability, treatment, competence, routine care, EAP, cuts, burnout, fragile teams, anxiety, stress, working hours, salary based, cheaper labour, inadequate care, increased working hours, workload,	Availability and suitability of healthcare staff, brain-drain to the private sector, burnout and stress increase	3) Competent, motivated, human resources	QS 7 Competent, motivated, human resources
machine, time restriction, all together, full room, inappropriate, fragile, un-practical, allowed, effort, breastfeeding, clock, pressure, conditions, influence, bathing, interruption practices, crisis, EAP, cuts, needles, preeclampsia, postpartum haemorrhage, birth, sepsis, premature	Adequate medicines, time restrictions, facilities, resources available, Capacity issues, closure of rooms, premature infections	Time and management of medical equipment, Capacity issues, Material and Equipment, Medication available	5) Essential physical resources available	QS 8 Essential physical resources available

^a^  Several examples were used, as not all concepts could be displayed

## Results

Our findings revealed that both healthcare professionals and experts perceived that the quality of care delivered to mothers and VPT/VLBW infants did not deteriorate during the implementation of the EAP in Portugal [*n* = 20/21]. According to participants, the extraordinary efforts of healthcare professionals and their resilience to the hardships encountered during this period prevented healthcare users from being negatively affected by the effects of the implemented austerity measures [*n* = 20/21]. In spite of the generalized perspective expressed by participants, the information of their accounts on their work experiences revealed that perinatal care was indeed adversely modified in all WHO Quality Standards [*n* = 20/21].

**Fig. 1:**
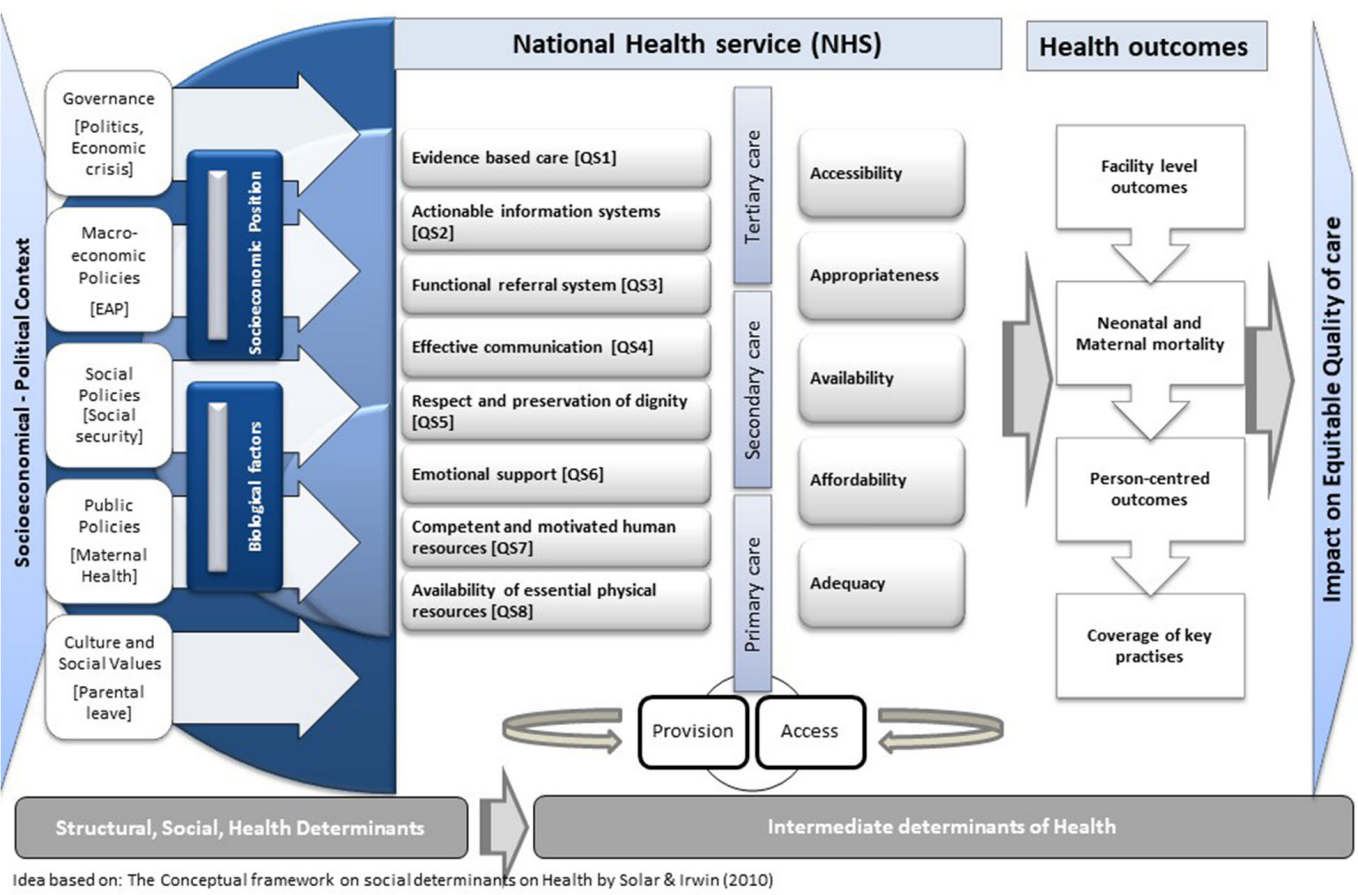
A conceptualized schematic representation on the macro-socioeconomic impact on perinatal healthcare quality for VPT/VLBW infants in Portugal. Author’s design

The perceived impact of macro-socioeconomic determinants on perinatal healthcare quality for VPT/VLBW infants is visualized in a conceptualized schematic representation (Fig. 1). The results were structured into the five main evolved themes: 1) Availability of Human resources; 2) Functional referral systems; 3) Competent, motivated, human resources; 4) Emotional support; and 5) Essential physical resources available. Additional quotes of participants are displayed in Supplementary material 3.

### Availability of human resources

This theme reoccurred as the main dominant and interrelated theme throughout the analysis. The reduction of human resources with prohibition of hiring and replacing of manpower losses in order to limit non-essential healthcare costs were perceived to have a direct negative impact on perinatal healthcare provision [*n* = 20/21]. These measures aggravated the unequal distribution of human resources and shortage of staff across different health care divisions [QS8] [*n* = 20/21]; deficiencies to adequately provide evidence-based care [QS1] [*n* = 20/21]; restrictions in the functionality of the gatekeeping-system [QS3] [*n* = 20/21]; incomplete operating teams [*n* = 14/21]; delays in admitting women for delivery, which caused potential high risks to mothers and infants [*n* = 6/21]; and deteriorated staff motivations [QS7] [*n* = 20/21]. Indirect consequences of the lack of human resources were brain drain of healthcare professionals to the private sector or other countries [*n* = 20/21]; early retirements [*n* = 16/21]; lack of young healthcare professionals [*n* = 14/21]; delays in timely care provision and examinations [*n* = 19/21]; and reduced emotional support for mothers with VPT/VLBW infants in NICUs [QS6] [*n* = 15/21].

*“Very delayed (appointments). Women who should have monthly consultations and sometimes are 2 months without getting consultations. […]”–* Informant 21

The financial cuts enforced on the healthcare system had mostly impacted primary care facilities. Lack of healthcare professionals and namely lack of GPs to provide routine antenatal and postnatal care was linked to several problems [*n* = 17/21]. It impacted provided care by limiting time management for healthcare professionals [*n* = 20/21]. Major shortcomings in routine care for pregnant women were: increased waiting times to schedule appointments [*n* = 19/21]; limited timely antenatal consultations [n = 17/21]; time-consuming waiting periods at the respective health facility when attending appointments [*n* = 15/21]; reduced number of appointments as GPs were unable to adequately respond [*n* = 19/21]; and failures in patient referral to specialists [*n* = 18/21] [QS1]. Number and frequency of antenatal consultations was indicated to vary depending on the clinical situation but also on the limitations of the healthcare centres [*n* = 18/21]. Overall time assigned to each consultation decreased [*n* = 15/21], especially at the first consultation during pregnancy [*n* = 11/21]. Participants reported that 86% of pregnant women at one primary healthcare unit, which serves one of the largest populations in Great Lisbon region, had not yet been assigned to a GP (family doctor) in 2018 [*n* = 3/21]. In another primary care units with around 50,000 users, it was again reported that nearly 50% of their patients did not have an assigned GP in 2019 [*n* = 4/21].

*“In relation to child health surveillance it was clear that people had to miss more surveillance appointments.”* – Informant 5

At hospitals, lack of human resources caused challenges in support provision for VPT/VLBW infants [*n* = 13/21]. It adversely influenced care provided by clinicians and nurses in neonatology and obstetric departments [*n* = 17/21]. Lack of advanced healthcare professionals implied for remaining staff: to have less time to provide suitable formation to younger colleagues [*n* = 6/21]; to be overworked [*n* = 20/21]; to be left with too few operating staff [*n* = 8/21]; and to be faced with persisting work pressure [*n* = 20/21]. When the EAP implemented frozen salaries along with a 40-week-hour schedule extended to nurse professionals, recurrent strikes due to discontent by nurses with increased working hours and decreased base salaries, have further delayed the admission of women with planned caesarean sections [*n* = 6/21]. Medical doctors were less affected in their base salaries because they were treated according to the rules of collective contracts. They were confronted nonetheless with frozen careers, decreased supplementary payments and higher workload to compensate for staff shortages [*n* = 16/21]. Brain drain of middle-aged clinicians from intensive and intermediate care units of public hospitals during that period triggered further time and management issues in the operating teams that continue to date [*n* = 13/21]. Due to the re-instalment of the 35-week-hour schedule in the post-troika period, the impact of shortage in nurses has been felt to be even higher after the crisis [*n* = 17/21].

### Functional referral systems

The lack of articulation between health information systems was classified as a restraining aspect for quality of care [*n* = 6/21]. Participants rated health information systems as ineffective due to serious deficiencies such as technological failures [*n* = 14/21] and poor articulation between actionable information systems [QS 2] [*n* = 14/21]. It was stated that the patient must either have had one prior consultation or been admitted to that particular health facility in order for healthcare professionals to be able to access past medical information and report exchange. The proper functioning of each healthcare facility information system depended on healthcare facility management [*n* = 5/21], human resources availability [*n* = 18/21], and user subscriptions [*n* = 4/21]. It was specified that actionable information systems did not function homogeneously across the country [*n* = 14/21].

*“Yeah, and when you look for indicators in more bureurifical [outer] regions, you’ll realise that the number [of health care units] drops drastically. So, the problem is not the number [of healthcare units] itself, it is the distribution. […] With the troika, the Government and policy makers realised that there was a need to cut public expending […] again, decisions became more centralised.”* – Informant 20

Geographically scattered and unequal distribution of primary care and hospital facilities was perceived to have obstructed timely access and adequate care provision for patients [*n* = 20/21]. Consequences for mothers were lower accessibility, increased inequalities in the availability of appointments, higher dependency on transport and longer waiting times [*n* = 15/21]. This has aggravated differential outcome and potential survival of preterm infants [*n* = 8/21]. The plan for the creation of reference centres by the EAP has not been completely implemented in all healthcare units until today [*n* = 11/21]. Moreover, an autonomously organized structure persisted in many units, which caused structural issues that have been impairing communication and coordination of care [*n* = 8/21].

Follow-up was perceived as a challenge [*n* = 18/21] due to: i) delays in follow-up exams on account of capacity issues [*n* = 9/21], ii) non-availability, incompleteness and incoordination of follow-up exams due to lack of specialized healthcare professionals (e.g., physiotherapy, social therapies) [*n* = 13/21], iii) incoherency in the gatekeeping-system as mothers without assigned GP were consulted by different clinicians in each consultation [*n* = 20/21] and iv) loss of referral continuity [*n* = 8/21]. The lack of coordination in follow-up care reflected prior crisis-existent structural and organizational issues of the gatekeeping-system [*n* = 15/21], which were then aggravated by insufficiency in human resources [*n* = 20/21]. Its deficiency hampered articulation and communication [*n* = 14/21] and homogenous functioning [*n* = 13/21] which impeded adequate tracking of the development of infants [*n* = 8/21]. It further occasioned uncoordinated and overlapped postpartum appointments in health facilities [*n* = 11/21] and ultimately over use of emergency care rooms [*n* = 19/21].

*“No, we don’t coordinate together. Everything is separated in terms of follow up.”* Informant 2

As already stated, the impossibility to hire additional staff during the EAP led to a disproportionate nurse- and medical doctor-ratio per patient [*n* = 20/21], particularly in primary care facilities. Regarding antenatal consequences, numerous mothers were not assigned to a GP which ended up in their exclusion from the referral system [*n* = 12/21] or in delayed referral to specialists [QS3] [*n* = 13/21] with consequences for diagnosis of potential complications for preterm birth (e.g., preeclampsia, diabetes) [*n* = 5/21].

The economic crisis directly influenced women with a lower socioeconomic status (SES) [*n* = 20/21] and the year 2010/2011 was pointed out as a social break point [n = 20/21]. Women with a lower SES, residing in areas distanced from main centres or deficient in health care provision capacity, attended perinatal consultations less frequently [*n* = 15/21]. Due to centralization and reallocation of care facilities, many pregnant women experienced accessibility constraints [*n* = 11/21]. Though pregnant women and children are exempt from NHS user fees [*n* = 16/21], they faced increased difficulties with time-consuming travel distances and transport costs [*n* = 14/21].

### Competent and motivated human resources

Lack of motivation, dissatisfaction and productivity loss due to the budget cuts and cost savings by the EAP were among the main identified issues [*n* = 18/21] [QS7]. Healthcare professionals in general earned less than 10 years ago [n = 16/21]. The 11% retirement deduction frequently led to the uptake of double shifts between public and private care [*n* = 16/21]. Principal demotivation factors pointed by participants were salary cuts associated with additional working hours (e.g., 12 h night shifts) along with a reduction in additional hourly payment [*n* = 19/21]. Additional stated reasons were: the reinforcement of contracts per hour “Recibos Verdes” with lack of employment benefits (e.g., disability income protection, annual bonus, extra hour and retirement benefits); withdrawal of working conditions such as paid meal provision during continuous shifts or personnel resting areas; and the lack of human and physical resources [*n* = 18/21].

*“I think the quality of care is still good, but with the cost of the health of professionals.”* – Informant 2

The EAP cost reduction measure included offering less stable hospital contracts and resulted in a less specialized and cheaper labour workforce which contributed to the fragility of working teams [*n* = 6/21]. Younger healthcare professionals were not sufficiently supported and faced issues in their career perspective [*n* = 4/21]. Young nurses indicated to have done their specialization aside from their work time for which they neither got time allocated nor were accordingly paid for [*n* = 4/21]. Young clinicians declared that they were often not hired after their specialization because there were no vacancies [*n* = 8/21].

*“What changed most was in terms of human resources and wages, as I was saying. It changes in terms of satisfaction, in terms of availability, in terms of burn-out, but not in terms of practice.” *–  Informant 6

**Fig. 2:**
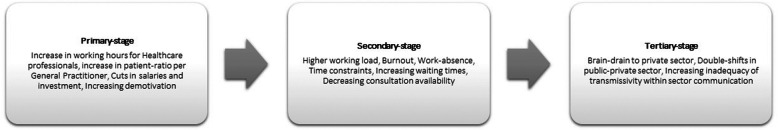
Stage-effect-chain

The reduction in human and physical resources while increasing working hours amplified their efforts to maintain quality of care at pre-crisis level but with a higher workload [*n* = 20/21]. The majority of healthcare professionals felt pressurized and overwhelmed with their work [n = 20/21]. It led to stress, burn out, 10% absenteeism at work, earlier retirements, and brain drain to the private sector or other European countries [n = 20/21]. The impact of the working environment of healthcare professionals was summarized in a three-stage effect chain (Fig. 2).

### Emotional support

Two major issues on parents support provision were described: i) insufficient formal postnatal support provision by the NHS, and ii) refusal of psychological support by mothers after birth. Psychological support after preterm birth was declared to be available on request [*n* = 11/21] and offered by at least one psychologist in the immediate postnatal period at the public hospitals [*n* = 15/21]. Healthcare professionals and experts claimed that frequently mothers did not remember that emotional support was offered to them as they were overwhelmed with the situation [*n* = 12/21]. Some mothers refused emotional support especially when birth was perceived as a traumatic event or when the infant was born with major physical or cognitive limitations [*n* = 13/21]. Parental leave for parents with VPT/VLBW infants was perceived as inadequate as it equals the support time of parents with a term born infant [*n* = 18/21]. Participants recommended to expand parental leave for parents with VPT/VLBW infants [*n* = 13/21].

*“In the early days of internment, the situation is so heavy that, often, though we offer support they refuse. And then they do not even remember that they refused. I think the situation is too intense, first, for us, professionals, to be able to judge it. […] they often do not remember at all that we’ve talked to them about this or that [...].” – *Informant 2

Higher stress levels among healthcare professionals and less available time to provide accurate explanations on care procedures, blocked effective interactions with staff [QS4]. It further inhibited information exchange between patients and healthcare professionals [QS5] [*n* = 17/21]. Participants considered that care provided did not always meet the needs of their patients and the required emotional support, which also led to greater demotivation [*n* = 19/21]. Healthcare professionals needed to prioritize their working time on mainly the immediate postnatal care (defined as the first month after birth) [*n* = 6/21] due to time constraints [*n* = 16/21]. Antenatal follow-up exams were either not provided in the obligatory frequency or to a lesser extent than mothers required [*n* = 7/21].

### Essential physical resources available

The EAP reduced equipment (e.g., computers) and stopped the substitution of technology to achieve resource savings that impacted significant essential physical resources [QS8]. Primary care units experienced higher material deficiencies compared to hospitals [*n* = 17/21]. Insufficient maintenance and replacement of equipment and medical necessities were pointed out [n = 17/21]. The shortage of material was defined as: i) lack of equipment and materials to carry out certain activities during the consultations [*n* = 19/21]; ii) outdated and slow-functioning computers computer systems that led to system failures and caused technical errors, significantly slowed the entry of medical data, and caused delays in consultations and examinations [*n* = 14/21]; iii) outdated or defective devices and technical resources (e.g., diagnostic devices, CATscan, MRT, ultrasound devices); iv) worn hospital beds; v) lack of simple tools (e.g., gloves, needles, paper); and vi) no resting places for parents and medical doctors [*n* = 18/21].

*“And the equipment that needed to be replaced, especially the ultrasound equipment, their approval was long overdue. The difficulties felt were in fact in the staff and the equipment. “* – Informant 4

Paradoxical and counterproductive problems due to lack of essential physical recourses were classified in peripheral hospitals [*n* = 6/21]. Even though deliveries have declined within the last decade, the number of nurses and medical doctors has also decreased due to the cuts of the EAP [*n* = 7/21]. VPT/VLBW new-born transferral was hampered because nurses could not accompany the inter-facilities-transport [*n* = 15/21]. It prevented the entry of preterm infants who needed an incubator or special treatment (e.g., hydrocephaly requiring neurosurgery or diaphragmatic hernia requiring cardiac thoracic surgery) and affected access to adequate care in NICUs [*n* = 13/21]. The transferral of infants to a HAPD without medical necessity increased [*n* = 14/21]. This was caused by frequent referrals of infants from HAP to HAPD because HAP did not have sufficient capacity [*n* = 14/21]. It led at times to the transferral of other infants who were in a slightly better condition from HAPD back to HAP [*n* = 17/21]. Participants recalled two situations in which infants were too early referred from HAPD back to HAP and deterioration in their health was observed [*n* = 4/21].

Capacity issues (e.g., lack of incubators, cots) led to the closure of available rooms with incubators at NICUs [*n* = 7/21]. Lack of cots and isolation spaces led to perinatal infections in one NICU [*n* = 6/21]. The lack of capacity in intensive care units NICUs and maternity units remained overcrowded and space in wards and beds remained deficient [*n* = 18/21]. Capacity problems were exacerbated due to the simultaneous ongoing closure of maternity units of under 1500 births per year in 2000–2012 [*n* = 7/21]. The need for larger intensive care units and intermediate wards [*n* = 8/21] and the recruitment of additional nurses at the HAPD [*n* = 20/21] was recommended.

*“ […] And we have a room [in NICU] closed because we don't have enough nurses. This room has been closed for 1-2 years. [...]. Even now, with the entrance of additional nurses we cannot open it, there are not enough yet.“ *–  Informant 6

Drug attainment has become cheaper since the EAP reinforced generics through prescribing the active substance instead of the commercial name [*n* = 21/21]. At the end of the Troika period, the generic market represented around 30 to 50% [*n* = 13/21]. The EAP also cut freely available therapeutic methods and birth control measures at primary care centres [QS 8] [*n* = 8/21]. Women with lower SES often bought only parts of medical prescriptions and rather chose the less expensive drugs [*n* = 8/21]. Participants indicated that pregnant women stated that they had stopped taking or limited buying certain medications as they could not afford all prescribed medicine due to a general inferior financial situation [*n* = 7/21].

## Discussion

Although participants did not perceive the quality of perinatal care as having deteriorated, the analysis of their responses on work experience revealed that it was indeed adversely modified in all eight WHO Quality Standards. Results disclosed an identifiable interrelation between macro-socioeconomic determinants and perinatal healthcare quality within all Quality Standards. High-quality care calls for appropriate usage of the healthcare infrastructure, skilled and motivated healthcare providers, adequate availability of materials, evidence-based clinical practices and non-clinical interventions to guarantee effective surveillance and organization [7]. Our results reveal that the major obstacle to deliver adequate care were the budget cuts in physical and human resources by the EAP which influenced the working environment of professionals and alternated perinatal healthcare quality.

Previous structural problems of the NHS were exacerbated by the austerity measures of the EAP. Overall, the unequal distribution of general practitioners and lack of GPs, nurses, obstetricians, and perinatologists created obstacles in perinatal healthcare. In primary care, the lack of GPs as key players of the gatekeeping-system caused obstacles in the functional referral as a result of a compromised gatekeeping-system with: higher waiting times, hampered timely access, and inconsistency in surveillance due to cuts in consultations, ineffective healthcare facility articulation and coordination, and incoherent follow-up. It ultimately led to inefficiencies and high budget spending on health as emergency care continued to be overused. In secondary care, lack of physical and human resources led to unnecessary paediatric inter-hospital transport of VPT/VLBW infants between HAP to HAPD. VPT/VLBW infants in need of special care and intensive surveillance due to prematurity complications experienced differential capacity issues in the referral system.

Healthcare professionals tried with extraordinary efforts to maintain quality of care under time constraints, high stress levels, and pressure to work with less essential physical resources. It caused less productivity, work absence, and ultimately burnout amongst healthcare professionals in primary and differentiated care facilities. We argue that this effort cannot be maintained over the long-term and will undermine sustainability of the NHS. Other studies confirmed diminished productivity, work absence, and burnout among Portuguese healthcare professionals, which was also associated with perceived poor working conditions and reduced professional experience [23–28] or crisis related reasons [29]. National statistics reveal that 21.6% of healthcare professionals indicated moderate burnout and 47.8% severe burnout between 2011 and 2013 [30].

Non-availability of human resources was further worsened through brain drain of healthcare professionals due the introduction of a 40-week-hour work schedule and salary freeze by the EAP [Act 68/2013 of August 29]. The decree-law [(updated) DL 71/2019 of May 27] particularly affected nurses which were hired by individual contracts, which faced increased hours of work with no extra hourly pay and no right to a day off between shifts. Many healthcare professionals left their position in public care and either changed to private care or emigrated to another country seeking better working conditions or chose an early retirement with severe salary cuts. An emigration wave of 12,500 nurses was estimated between 2009 and 2015 due to: worsened working conditions through salary cuts, the search for better working conditions, financial problems caused by the crisis, and a demoralized workforce [31, 32]. In 2016, a study reported that 15% of medical doctors considered migration due to alternated working conditions by the crisis and EAP (e.g., 30.5% decrease in public compensation), and disclosed a higher demand of health care provision, a decrease in public care and an increase in private care [33]. The 35-week-hour schedule was re-established in 2016 [Act 18/2016 of June 20] but only applied for individual contracts at public services in July 2018. In 2019, 8 years after the onset of the crisis, the lack of nurses due to that re-instalment was experienced to a greater extent than during crisis period (2009–2016).

The shortage of human resources was tried to be restored and reorganized by: i) providing an extension of GP patient lists from 1500 to 1900 patients per GP in 2013; ii) hiring 2000 healthcare professionals between 2013 and 2014 to reduce 50% of the shortage from 1 million to 5000 GPs and; iii) implementing a family nurse in 2014 [Decree-Law 118/2014 of August 5] [34]. Still, the shortage of human resources remains a major challenge for all cross-sectional services of the NHS until today [35]. In 2015, the NHS employed 4.6 GPs and 6.3 nurses per 1000 patients, compared to the EU average of 3.5 GPs and 8.4 nurses [36]. Even though the GP provision is slightly above and nurse provision below EU average, it is arguable which ratio would be best to achieve a high-quality care provision in a country-based context. Given the unequal geographic distribution of GPs, national statistics on emigration and burnout, and the responses of participants, the availability of doctors and nurses who could provide care persists as inadequate.

The EAP sought to increase the number of primary care units operating under regional government contracts with a mix of salary and performance-related payments in order to be more autonomous and to establish a mechanism to ensure a more even distribution of GPs across the country [37]. However, it was found that unequal geographical distributions of health facilities have continued until today [38]. As of the end of 2017, there were still 390 non-reformed family health units compared to 505 reformed ones, of which around 235 received a performance-based allowance [39]. Enhancing accessibility to primary care has not been fully achieved by the EAP and the population without a GP across the country remains high [23, 26]. Other studies also revealed that longer travel distance due to the lack of nearby facilities was a major factor in the increase in emergency visits [40]. Geographic access continues to be one of the major challenges in accessing health care between low and high income groups and health care facilities remain unevenly distributed [41, 42]. In 2017, Portugal had 225 hospitals, of which 107 were public, 114 private, and four public-private partnership hospitals. The majority [*n* = 208] was located across mainland Portugal [43]. Primary care centres, which are obliged under the Basic law [Act 95/2019 on September 4] to be allocated in the immediate vicinity at regional level, have been so far mainly concentrated in the main metropolitan area of Coimbra (26%), Lisbon (25%), and Porto (24%) [42]. EAP reforms have also potentiated the rapid growth of the private health care sector which surpassed the public in number of facilities in a few years. Their services are, however, only accessible as long as paying users can afford it.

The fragility of a crisis-affected population was among other reflected in the medication intake behaviour of users, which was verified by other studies [26, 43–45]. In 2011, the EAP introduced several measures on drug purchasing: i) setting the maximum price of the first generic presented in the market to 60% of the branded product; ii) user charges for over-the-counter drugs; iii) reinforcements in generic prescriptions which reached 40% in 2013; iv) and compulsory electronic prescription for medicines and diagnostics covered by public reimbursement for medical doctors in public and private sectors [37]. The EAP introduced changes in user fees which reflect the not absolute gratuity of the NHS: i) reviewing existing exemption categories (e.g. pregnant women and children under 12 years); ii) extending co-payments for most services; iii) and increasing user charges [37]. Even though the EAP substantially reduced the prices of medications and pregnant women are exempt from user charges, participants reported that mothers of VPT/VLBW chose among the cheaper medications on the prescriptions in 2018/2019.

The crisis and EAP especially influenced pregnant women with lower SES in a threefold manner which resulted in lower or non-attendance of antenatal consultations at primary care centres. Firstly, patients faced inferior monetary situation and unemployment due to the crisis [33]. Secondly, the EAP reduced one-third of patient transportation by limiting non-urgent patient transport and implementing detailed rules for health service provider on transportation authorizations which diminished free transportation [32, 37, 45, 46]. Thirdly, economic and financial crisis effects influenced behaviour of women who postponed their maternity as another study confirmed [39]. Non-attendance of consultations caused issues in patient referral, information provision, communication, support during the antenatal and postnatal period and overlap in postpartum appointments. Despite exemption from out-of-pocket payments, intensified monetary hardship through decreased household income consequently adversely affected healthcare access for pregnant women.

Decreases in infant mortality rates, commonly used as a measure of population health and quality of health care when considering healthcare outcomes, represent an enhancement in socio-economic conditions and quality of obtainable health services [47, 48]. In 2017, 1.8 per 1000 live births in Portugal compared to EU-19 average of 3.3 were reported [10]. The crisis was associated with a significant increase of low birth weight rates in Portugal between 2008 and 2011, resultant of health expenditure decline, slowdown of general gross product (GDP), and increased unemployment [49]. The study indicates that it was mainly caused by reductions in government expenditure on health as a proportion of GDP and reduced percentage expenditure of social protection and healthcare [49]. When looking at perinatal deaths, a slight increase in perinatal deaths with 2.9 to 3.6 per 100 live births was recorded after the crisis hit Portugal between 2010 and 2012 [3]. Similar observations were made in other crisis-affected European countries which reported effects on health within the same time period. In Greece an increase in infant mortality rates of 43% and a significant rise in the proportion of low birth weight and stillbirths, and in Italy a significant drop in fertility rates was observed [50]. Recent statistics of November 2019 revealed that due to the consequence of pregnancy complications, maternal mortality was at 17 women per 100,000 births in 2018, compared to 9 women per 100,000 births in 2017 [51]. This retrospectively corresponded to the same values described in 1980 with 19.5 maternal deaths per 100,000 births [51].

The adverse impact of the recent economic crisis on healthcare system provision and health service utilization has been widely discussed [24, 32]. European wide, vulnerable populations such as children or pregnant women were one of the first groups to be affected from economic hardship and to have suffered from health inequalities [52]. Common impacts were the increase of differences in access due to higher financial burden to household, the reduction of adequate response to health needs, and the decrease of satisfaction with health services [33, 45]. Since the late twentieth century, privatization in healthcare and the reinforcement of the free market system has been at the forefront of political agenda and applied as a shared principle in countries facing rapidly rising health care costs and decreasing public resources [48]. Rising healthcare costs and high economic burden have been commonly addressed with the application of austerity policy and privatization aiming to save non-essential healthcare costs [53]. However, consequences of privatization have been linked to the intensification of health inequalities in accessing healthcare due to reduced availability of public financial resources for health service coverage and investment [23, 45, 54]. Despite its greatly political debated controversy, public health response on the impact of austerity measures on provision and accessibility of health services has been scarce [55].

### Limitations and added value

A limitation depicts a relative low generalizability of study findings due to the nature of a qualitative study. Yet, as our study focused on the two main metropolitan areas of Portugal where the majority of health care units are concentrated, the findings are still of high importance and partially generalizable. A minor limitation is that the participants might have been more susceptible towards the study as they communicated their interest and availability.

The added value is the disclosure of an in-depth understanding on the interrelation of macro-socioeconomic determinants and healthcare permitting a distinct representation from quantitative methods. The non-linearity between policy response and expected outcomes chiefly complements its comprehension and demonstrates its relevance for further research on assessing effects of austerity measures.

## Conclusion

Though the quality of provided perinatal care was not perceived by healthcare professionals and experts as having worsened, the analysis on the accounts of their work experiences revealed that it was indeed adversely modified in all WHO Quality Standards. The EAP was perceived to have directly influenced the working environment of healthcare professionals through budget savings and austerity measures causing stress, burnout, work absence, and deficiency in human resources in Portugal. Modified equitable perinatal healthcare quality through deteriorated timely care provision, increased waiting times, access inequalities, cuts in consultations, and lack of follow-up care for VPT/VLBW infants and their mothers was disclosed. The crisis and the EAP were evaluated to have particularly adversely affected mothers with lower SES through economic hardship which influenced their behaviour on accessing healthcare facilities and medication intake. Differential vulnerability and exposure to ill health in the long-term was aggravated amongst social groups. These findings underlined the impact of austerity policies on vulnerable populations.

### Recommendations

Firstly, we would recommend a higher focus on the inclusion of social policies into health policies in order to mitigate the effects of the economic crisis and the EAP. Secondly, we would consider the prolonging of the exclusive maternal leave period as predominantly necessary to strengthen maternity protection and encourage motherhood in a cost-effective way. Thirdly, we recommend that greater attention should be placed on the equal geographical distribution of primary care facilities to allow timely antenatal care and perinatal screening possibilities. Finally, a greater transparency and equity on regulations and professional wages between the private and public sector would maximize quality of care and balance human resources distribution throughout the health care system.

## Supplementary Information

**Additional file 1.**

**Additional file 2.**

**Additional file 3.**

## Data Availability

The datasets generated and/or analysed during the current study are not publicly available due to data protection and data privacy which is in accordance with ethical clearance and signed informed consent provided by participants which guarantees their anonymity and confidentiality, but are available from the corresponding author on reasonable request.

## Abbreviations

EAP: Economic Adjustment Programme
EU: European Union
GA: Gestational Age
GDP: Gross Domestic Product
GP: General Practitioner
HAP: Hospitais de Apoio Perinatal (Perinatal care hospitals)
HAPD: Hospitais de Apoio Perinatal Diferenciado (Differentiated perinatal care hospitals)
NICUs: Neonatal Intensive Care Units
SES: Socioeconomic status
VPT: Very preterm
VLBW: Very low birth weight
QS: Quality Standards
