# Supplementary material for: Impact of macro-socioeconomic determinants on sustainable perinatal health care in Portugal: a qualitative study on the opinion of healthcare professionals and experts
Source: BMC Public Health. 2021 Jan 25;21:210. doi: 10.1186/s12889-021-10194-0 (PMC7836450; doi:10.1186/s12889-021-10194-0)
Supplement: Supplementary file 1 — Additional file 1. [file 12889_2021_10194_MOESM1_ESM.docx]

**INTERVIEW GUIDE**

**Impact of** **macro-socioeconomic determinants on sustainable perinatal healthcare in Portugal:** **A qualitative study on the opinion of healthcare professionals and experts**

**CONTEXT**

We are preparing a new study which is coordinated by the principal investigator Professor Henrique Barros. This study is funded by the European project RECAP preterm which has received funding from the European Union Horizon 2020 research and innovation programme under grant agreement No 733280. The study falls under the general objective of the project to improve the health and quality of life of children and adults born preterm.

**ABOUT THIS STUDY**

This study is called **"Impact of macro-socioeconomic determinants on sustainable perinatal healthcare in Portugal: A qualitative study on the opinion of healthcare professionals and experts”** It intends to explore the impact of macro-socioeconomic determinants, such as the economic crisis and the Economic Adjustment Program (EAP), on the quality of healthcare for very preterm/very low birthweight infants in Portugal. The Economic Adjustment Program (EAP) was implemented by the Portuguese government in 2011 as a response to the economic crisis. The EAP introduced health care reforms and austerity measures to the National Health Service.

**MAIN OBJETIVE**

This study aims to analyse the impact of the economic crisis and the EAP on perinatal healthcare provision quality for very preterm/very low birthweight infants as perceived by healthcare professionals and healthcare experts, within the health administrative regions of the two major metropolitan areas in Portugal.

**METHOD**

This study applies a qualitative methodology using semi-individual interviews. Participants include healthcare professionals, who have work experiences in the field in public and private care during the pre-, and post- implementation period of the EAP, and health care experts who are involved in policies and decision making during the same time period and have expert knowledge on the topic.

Therefore, we would like to obtain your contribution through your participation. Main researcher Julia N. Doetsch and researcher Sandra C. S. Marques will conduct the interviews. The interview can be held on any day and at the place of your preference. The interview lasts around 45min - 1 1/2h. It will be audio recorded if you grant permission.

**CONFIDENTIALITY AND PRIVACY**

Your identification will be kept anonymous for privacy reasons. This study and all its investigators involved are subject to comply with the General Data Protection regulation (GDPR) [(EU) Regulation 2016/67]. The GDPR requires that all necessary privacy measures are implemented to ensure data storage security, confidentiality, and anonymity. The national standards and specific requirements of the Ethics Committee involved as well as data protection authority are met.

**INFORMED CONSENT**

We ask you to sign a written informed consent before the interview. This consent will include information on the terms of your rights for data privacy, data storage, and request of data insights and removal. It will be handed out to you in a printed form on the day of the interview. You will remain with a copy of the consent form to execute your data protection rights and requests at any time.

**ABOUT YOUR PARTICIPATION IN THIS QUALITATIVE STUDY**

Your participation is voluntary. You can respond to the questions that you feel comfortable with. The continuation of the study is not mandatory. If you wish, you can stop participating at any time, without any disadvantages for you.

**CONTACT DETAILS**

For any questions or further clarifications, please contact the above-mentioned researchers. Their contact details are in your copy of the informed consent statement that was attached to this information.

**MAIN OPEN-ENDED QUESTIONS**

**A) ECONOMIC CRISIS**

1. Did the economic crisis influence the Portuguese health system and its users in your opinion?
2. Has there been an impact of reorganization and centralization policies on the quality of care, which were implemented before the crisis? (e.g. merging of public hospitals)
3. What is your opinion about the interaction between the private and public sectors in the Portuguese health system?

**B) ECONOMIC ADJUSTMENT PROGRAM (EAP) [2011-2014]**

1. Was there an impact of the EAP, which was implemented in response to the crisis, on health care in the NHS?
2. Do you think that the EAP measures had influenced or are still influencing healthcare performance and healthcare provision?
3. Do you think there were positive or negative effects of the EAP for the NHS?
4. Do you think that EAP influenced positively or negatively the performance of healthcare professionals?
5. Do you think the goals of the EAP have been met?

**C) HEALTH CARE PROVISION AND USAGE**

1. Do you think that the economic crisis and the EAP influenced the usage of the health services for patients?
2. Do you think that the economic crisis and the EAP influenced the provision of prenatal or postnatal care in terms of prevention, monitoring, or therapy?

**D) POLITICAL PRIORITIES**

1. What are the challenges of providing health care to premature children in Portugal? (e.g. parental leave)

**E) RECOMMENDATIONS & COMMENTS**

1. What are your recommendations for improving health care delivery for parents with premature children?
2. Would you like to add something that was not asked?

Thank you for your time and consideration!
