# Supplementary material for: Impact of macro-socioeconomic determinants on sustainable perinatal health care in Portugal: a qualitative study on the opinion of healthcare professionals and experts
Source: BMC Public Health. 2021 Jan 25;21:210. doi: 10.1186/s12889-021-10194-0 (PMC7836450; doi:10.1186/s12889-021-10194-0)
Supplement: Supplementary file 2 — Additional file 2. [file 12889_2021_10194_MOESM2_ESM.pptx]

## Slide 1
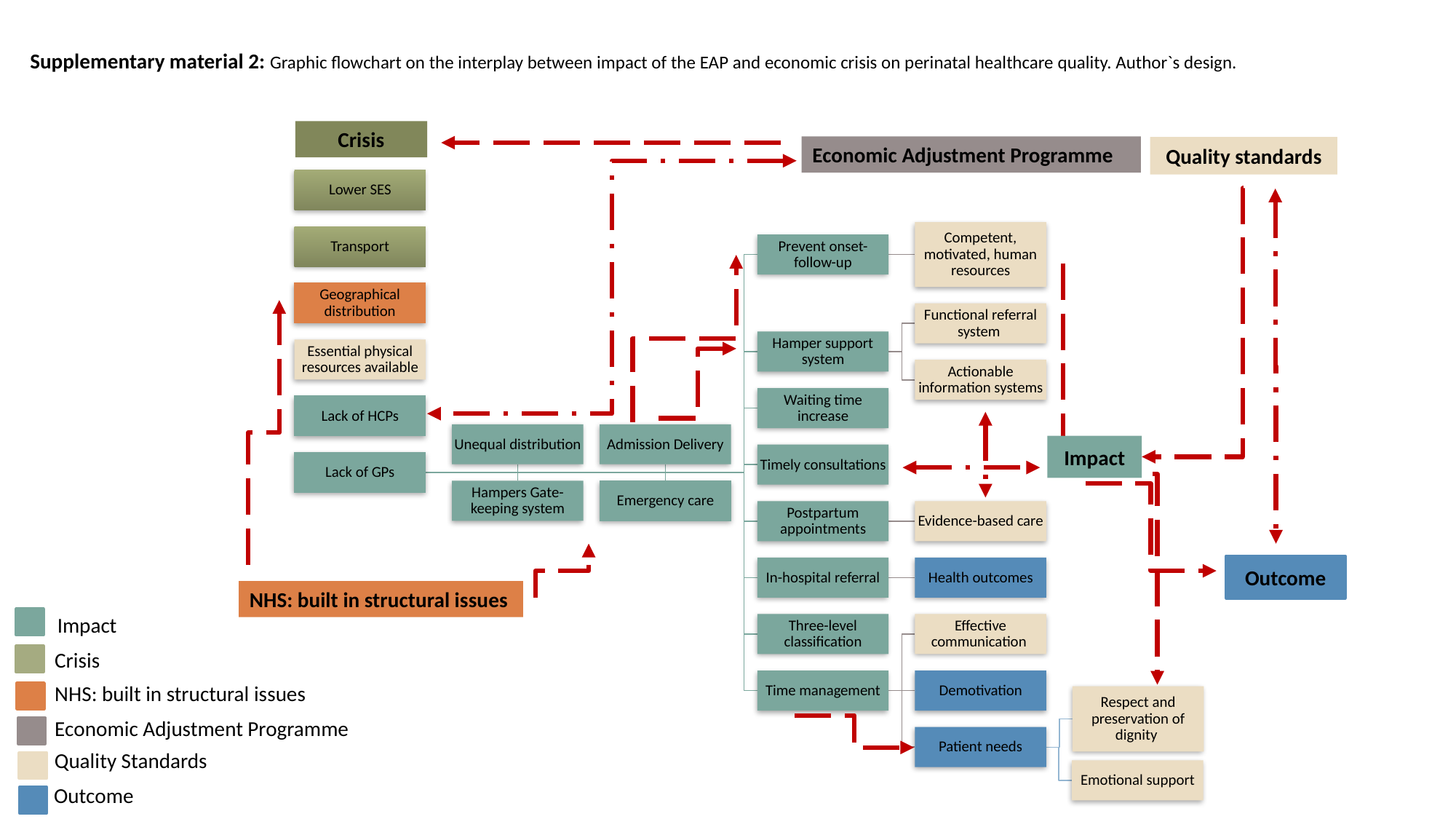

Supplementary material 2: Graphic flowchart on the interplay between impact of the EAP and economic crisis on perinatal healthcare quality. Author`s design.
Crisis
Economic Adjustment Programme
Quality standards
Impact
Outcome
NHS: built in structural issues
Impact
Crisis
NHS: built in structural issues
Economic Adjustment Programme
Quality Standards
Outcome
