# Supplementary material for: Impact of macro-socioeconomic determinants on sustainable perinatal health care in Portugal: a qualitative study on the opinion of healthcare professionals and experts
Source: BMC Public Health. 2021 Jan 25;21:210. doi: 10.1186/s12889-021-10194-0 (PMC7836450; doi:10.1186/s12889-021-10194-0)
Supplement: Supplementary file 3 — Additional file 3. [file 12889_2021_10194_MOESM3_ESM.docx]

| Supplementary material 3: Additional quotes from Participants | |
| --- | --- |
| Content Analysis Themes | **Quotes** |
| 1) Availability of human resources | |
| *Primary care provision* | |
|  | “It’s like 500 000 people still waiting for the family doctor. […] [before it was] about 1million people without family doctor. So we tried to recover and we recovered fast in 2 ½ years. But I am not sure what will happen in the upcoming years. Because there was another change. The troika […]; the patient lists increased from 1500 to 1900 which still remains nowadays. Not only we need to provide family doctor to those 500 000 people, but […] to reduce again the list from 1900 patients per doctor to 1500. **Informant 20** |
|  | “We have the structural problems right from the beginning of the NHS […]. And in terms of distribution in the territory. [..] most professionals are located in the urban areas, Lisbon, Porto, Coimbra. […] The decrease in the salary and the allowance to enter more professionals was frozen [EAP measure], so it was not allowed to replace, to hire new professionals.” **Informant 20** |
| *Secondary care intra- and postnatal care provision* | |
|  | “The crisis goes on in the health system until today. […] for example, with the change from 40 to 35 hours […] we have less hours of working nurses than we did within the journey of the crisis time. So, we actually have fewer human recourses than we did during the troika. And even the investment that has been made with hiring more people is inefficient to compensate people and the number of hours we`ve lost. When we look at waiting times for example, we have recommended waiting times. And they are rising every year. So, one can say that theoretically we are out of the crisis, but the crisis has not left the health system.” **Informant 18** |
|  | “No, it didn’t solve the issue. It was just a stressful situation, ok? We cannot pay the debt, we cannot pay the expenditures, we have to cut. So troika was cutting everywhere, cutting the wages, cutting the professionals, cutting the beds, cutting everything. But then no real structural change in this situation, ok? And at the same time a strong development of the private sector puts a lot of pressure on the public sector. I don’t think that the troika, even if I think that there were good points in that period, successful in the case of drugs, kind of successful in other kinds of initiatives, but it was not a good period. I don’t think that this period of austerity was good for the country. That for sure was not the case. And for sure it was not good for the public-system.“ **Informant 16** |
|  | “When the first batch of nurses left the [name of hospital] [HAPD in Lisbon] it was scary. Either services were closed or nurse staff had to work overtime. Now this policy of overtime is extraordinary in itself! How is it possible to have a scale of work done with extraordinary work at the outset? This is weary for the team and leads to a certain point when no one is able to do more than the minimum required, only the minimum is met.” **Informant 14** |
| *Waiting times and time management* | |
|  | “[…] they have to be given enough time to know the patient, so that the patient feels "taken care of". […] it is part of "caring" to have time to listen more. It is part of the cure ...”. **Informant 11** |
|  | “[…] some main hospitals have unit-based follow-up of the child development. I don`t think that all the hospitals have. […] I think the major problems are the follow-up and the problems that we have regarding physiotherapy, social therapies … all the therapies that babies that have handicaps need to have. Because although the Government and the healthcare division says that we have everything to provide to those babies. And […] that they are well-coordinated, we see that this is not the rule. […] in general, they are not well-provided. That is the main problem […]. There is, I think there is a huge lack of specific care and there is a lack of experience on the long-term problems of prematurity. […]. In terms of physiotherapy, speech therapy, occupational therapy, psychotherapy and all that.” **Informant 1** |
|  | “In Maternal Health and Family Planning, maternal health care does not accompany risky pregnancies. From the moment we identify risk we refer to the referral hospitals. It was the consequences of the crisis. Lack of jobs. Basically. It’s like that, at the time of the economic crisis everything diminished. There were fewer resources, we only had [name] hospital which was full of users everywhere, huge waiting times. We referred a pregnancy for urgent consultation and she was waiting a month or more. And when I say urgently, it was really a reference made by us as urgently required.” **Informant 21** |
| 2) Functional referral systems | |
| Lack in communication and articulation | |
|  | “The legislation exists and it was recently adjusted, but its implementation fails. We have a whole constellation of structures supposed to interconnect, but in practise, it doesn’t work well. It doesn’t work well or the structures are overwhelmed by an excessive number of children needing these supports.” **Informant 11** |
|  | “We have old computers and the computers work slowly […]. I think we have too much information in the computer, some programs and sometimes it’s not easy [to work with it]. And they give us small space for working. 1 GB for the whole unity and sometimes it’s really slow.” **Informant 2** |
| *Non-attendance of antenatal care consultations* | |
|  | “I think so. It [the crisis] influenced a lot. Personally, in our area, I can say that since maybe 2010. So, I've been here for some time. We know this is a population with a low socioeconomic level. And during and after that time period, in 2010 or 2011 we noticed that there was a huge social break. In Maternal Health and Family Planning, maternal health care does not accompany risky pregnancies. From the moment we identify risk we refer to the referral hospitals.” **Informant 14** |
|  | “we needed to comply with a set of measures we had agreed on. And of course, healthcare was one of the main issues. And I would say once again the financing of the system. We ended up of having a billion Euro cuts, per year, out of the 10 billion budget. […] So, people in general, citizens of this country were under a lot of pressure, financially, and therefore that has consequences in terms of their own health. A lot of people was even in more need of health care and more dependent on the public system, that of course needed to respond. And sometimes it was unable to respond as well. Theoretically we have ended the crisis in 2014, and we ended the troika intervention. But if we look at the data, all of these issues, the crisis goes on in the health system until today.” **Informant 18** |
| *Referral system articulation* | |
|  | “I do not agree that something has resulted from this [EAP] in practice. The only thing I see is that: it is not spent because it is not available. Therefore, people look for some alternative. We [Portuguese] continue to go to the emergency room instead of going to primary care. We have a reality here: […] From the study we did, in 2018, out of all the pregnant women who signed up for the first time to begin their surveillance process, 86% of pregnant women were without a family doctor ... What would be possible to improve in terms of indicators? I do not believe that out of the strategies defined, 80% had come to realisation.” **Informant 13** |
| *Shortage in staff and capacity in inter-facilities transport (TIP)* | |
|  | “We get a vacancy, later they get better and they can go home or else they may still need care at the peripheral hospital. To transfer this child in need of hospital care, one of our nurses has to go with the child during the transference to the hospital. Sometimes, I say, look, you can go, there is a vacancy. But the nurse tells me, "Doctor, I can only make one nurse available to accompany the transfer next Thursday!" […] Therefore, the child has to stay another week in this unit, in a central hospital, where the daily rate is very expensive, without need, and far from the parents’ home, because we do not have one person available to go with the child! And we are talking about intra-hospital transport that is only for serious cases […].” **Informant 3** |
|  | “[…] sometimes it is difficult to accept all the babies, and so sometimes we have to transfer them for other places. So I feel that our centre should be bigger with more intensive care units. The intermediate care units should be more also. Because when babies are ok, they passed the intensive care and they have to get for instance alimentary autonomy or they are only with antibiotics but they are stable, at that time they need intermediate care. And it was important that intermediate care would be here and not somewhere […]. What we do, sometimes we send them to paediatrics, paediatrics is not ok, the paediatric internment is not good. Sometimes we transfer for the hospitals of the second level, near the place where the parents live, but we feel that we have few units of intensive care and very few units of intermediate care too.” **Informant 9** |
|  | “The big problem was when the fusion of the hospitals occurred, 6 or 7 years ago, so 2011 or 2012. […] and when they decided to fuse, they have not done very well the things. Because some departments were better here, and there were some departments which were much better there. And they didn’t make any assessment about what was the best.“ **Informant 2** |
| *Lack of coherent follow-up and coordination* | |
|  | There is no linkage in the process of observing, being followed, give the results and, in some days, someone is responsible for you. The organization is lacking. […] It’s complicated.” **Informant 11** |
|  | “If the doctor who observed the child here does not consider that should be referred to hospital paediatrics, we can refer to early intervention, but they, at the ELI, are taking 2-3 months just to contact parents for the first time because they are not able to respond at all to the high demand. […] …or we decide that the child should be referred for Early Intervention and we make the request. But they have many children, take long time to respond and it is limited… […]. They are here in Continuing Care services. [I say they, but here, they have] one person. […] They hold their meetings and then forward where they can… they also have connection with hospital care**.**” **Informant 8** |
|  | “Yes, they said it’s [the gate-keeping system] the best thing to do of having the primary care seeing all the patients in that area. And one percent only goes to the emergency. But it’s not the case. The emergency room is always full. Because the primary care works only until some hour. [Plus] they don’t have the clinicians [GP`s] to attend to the patient’s needs.” **Informant 11** |
|  | “For example, in this [primary care] unit, that is the largest in the country, it is not possible to guarantee the family doctor, and after that, therefore, it is very complicated to follow the children, the pregnant women and the minimum surveillance plan that the State says it guarantees! Maybe because I also came to a UCSP unit. It is the [kind of primary care] units that are not organized in UCSF and most of the users here do not have family doctor.” **Informant 12** |
| \| 3) Competent, motivated, human resources \| \| \| --- \| --- \| \| *Motivations and burnout* \|  \| \|  \| “We already talked about it: salary cuts, increased working hours, doctors are asked to do more [emergency shifts]. So, these all lead to burnout. People are tired. The burnout really happens. I have felt it so many times. I have never had a depression or had this tendency, but I've been exhausted many times. And we have family too, and we can't take care of so many things. **Informant 8** \| \|  \| “There were cuts! And yes, just to improve state budget management, Ok! For our part, it translated into 40h working hours with the decrease in wages. Unemployment, with doctors and nurses hired by mediators. Either they went abroad or subjected themselves to these companies; the nurses at some point were paid at 4 € per hour.” **Informant 15** \| \|  \| ”What changed most was in terms of human resources and wages, as I was saying. It changes in terms of satisfaction, in terms of availability, in terms of burn-out, but not in terms of practice.” **Informant 6** \| \|  \| “Exactly. Just having two or three people on medical leave, it means that other people have to compensate for them. In the old days, people compensated because overtime was well paid. Tired but compensated. Now it is almost the person who pays to come to work and not the other way around.” **Informant 5** \| \|  \| “If I think that the EAP influenced the performance of health professionals, salary cuts, etc.? Of course, yes. I, for example, took a gross cut in my salary! And the increase in working hours was also something else that I was angry about. Not because of the increase in working hours, I do not see that the 40 hours were bad, but because they have to be paid! We cannot tell a person who is hired to work 35 hours on a pay-check X, who suddenly works more hours and the pay-check is the same. This is "outrageous" pure and simple. And if, in fact, those hours were paid, there wouldn’t be the strikes that existed and so on.” **Informant 4** \| \|  \| “Increase of working hours… during the troika and maybe we still feel that one. They are not admitting people, so we have to work more. We are still not feeling that we are working less. Because if we are not so many and some people are leaving the hospital. You have to work more. Ad there is another thing, more hours and you have more tasks. You have more things to do. And you are feeling that you are not doing it so well sometimes.” **Informants 7** \| | |
| 4) Emotional support | |
| *Psychological and formal support provision* | |
|  | “The mother can interrupt the maternity leave during the period the baby is hospitalized. But the state only pays the mother the four months of their monthly salary [of leave that any mother is entitled].” **Informant 1** |
|  | “For example, parental leave for premature infants, as far as I know, I think parents can ask for another month for child care. Maybe for a very premature this is not enough. Because they stay in hospital a long time…” **Informant 7** |
|  | “Actually, in intensive care, the ideal is sometimes a nurse for a baby, 1: 1. Which is hard to achieve. But if we didn't have the crisis, would it be much better? I can't be sure about this, can I? With this nurse cut and the 35-hour reversal, it was also very complicated to make their schedules, wasn't it? If there had been no economic crisis, would we be better? It's possible. But I don't know either.” **Informant 6** |
|  | “It influenced. It influenced both the economic crisis, firstly with restriction of the ratio health professionals/ inhabitant; we are less, the ratio is different than it was and the needs were for more nurses, more doctors, in many places, and secondly the reorganization in health care.” **Informant 15** |
| 5) Essential physical resources available | |
| *Capacity* |  |
|  | “With these restrictions and with the EAP, it was not possible to hire so many doctors; perhaps not enough infrastructures have been created. At the hospital level you do not feel so much. And I even think there were hospitals that were already under construction. But in the units of the community I think it fell short. **Informant 4** |
|  | “Maybe the only thing I can say is that if there had been no crisis… we needed to have a much larger ward, it's tiny and moms sometimes have to get up for me to get to the incubator. […] We have 8 intensive care incubators, and right now we have 10 intermediate care incubators….“ **Informant 6** |
| *Material and Equipment* |  |
|  | “In child health, I think that was more noticeable was the breakdown of vaccine stocks.” **– Informant 15** |
|  | “Yes, and what I’m saying is that are things that are still happening at the moment. So, the loss in autonomy, the possibility to hire, the impossibility to invest, started during the crisis but it’s now going on. So we have still huge problems related to that period. We are feeling and paying the consequences of that period. And so, the hospitals they are in a very bad financial situation at the moment and this started also during the recession. So, this is for the public sector. So we are now paying the consequences of that. So, the pressure on the workers became stronger and stronger, but they went on doing what they did before. And the other advantage what happened during the crisis is that the prices of the pharmaceuticals decreased, so the hospitals were still able to buy drugs. So, there is no major event of cuts in the provision of drugs for the patients or in the provision of surgeries.” **Informant 16** |
|  | “The authorization [for equipment replacement] only came 1 year ago, 2 years after I left! “Informant 4 |
| *Medication* |  |
|  | “The fact that the population has become poor; that the unemployment has increased. This all has consequences for the people. So. the increase in the usage of consultations and hospitalizations, I guess it is related to the deterioration of the living conditions of the patients. The strongest evidence we have on this is about the mental health. […] So, at this time that the public system was experienced the cuts and the need of the population increased, so it is a different situation.” **Informant 16** |
|  | “One thing: it seems there is a contradiction. You think that the troika did well in regulating the drug market, so how is it possible that users feel difficulties in accessing medicines? It is not a matter of the costs of the medications, it’s a matter of people having lack of money. And I need to start selecting their basic needs and even if the box of medication costs 5 Euros, those 5 Euros might mean that people don’t have money to eat that day. It’s not the costs of medications itself - which was reduced, and I think that it is at the bottom that it can be reduced – Probably, it’s a question of the money available for people to manage their daily life. **Informant 20** |
